# Carotidynia: Nonsteroidal Anti-inflammatory Drug Treatment Resulting in Symptom Resolution Within Weeks

**DOI:** 10.7759/cureus.80475

**Published:** 2025-03-12

**Authors:** Frederica H Ferreira, José Pedro Manata, Paula Cerqueira, João Matos Costa

**Affiliations:** 1 Internal Medicine, Hospital Distrital de Santarém, Santarém, PRT; 2 Internal Medicine, Hospital Distrital De Santarém, Santarém, PRT; 3 Internal Medicine, Unidade Local de Saúde do Alto Minho, Hospital Conde de Bertiandos, Ponte de Lima, PRT

**Keywords:** atypical neuralgia, carotidynia, nsaids, periadventitial thickening, recurrence

## Abstract

Carotidynia, also known as Fay syndrome, is a rare vascular disorder characterized by continuous or intermittent lateral cervical pain in the region of the carotid artery bifurcation. It can be associated with various vascular and non-vascular processes and typically includes the presence of periadventitial thickening, with preservation of the lumen and without causing hemodynamic alterations.

We present a case of a 45-year-old woman who was referred for an internal medicine consultation due to the presence of a foreign body sensation in the right cervical region. A CT scan of this area revealed unilateral perivascular thickening of the right internal carotid artery. A benign course of naproxen, a nonsteroidal anti-inflammatory drug (NSAID), was initiated, resulting in symptom improvement. Three months later, a follow-up CT angiography for vascular monitoring was performed, which showed significant improvement in the thickening around the right internal carotid artery.

Carotidynia has been found to respond effectively to NSAIDs or corticosteroids, with resolution typically occurring within weeks following treatment. However, it is important to note that recurrences are possible, especially due to poor adherence to treatment.

## Introduction

Carotidynia or Fay syndrome was first described in 1927 as an atypical neuralgia of the face and neck [1]. It is a rare vascular disorder characterized by continuous or intermittent pain in the lateral cervical region at the level of carotid bifurcation, which in some cases radiates to the facial and/or auricular ipsilateral region. It is considered by the International Headache Society (IHS) as a variant of migraines and is treated as such [1]. Recent publications show it is a distinct pathological entity with specific structural changes. Studies using ultrasound/Doppler, MRI, and magnetic resonance angiography reveal circumferential or eccentric enhancement of the arterial wall, limited to the adventitia, with preservation of the arterial lumen. This periadventitial thickening is histologically characterized by the presence of non-specific inflammatory changes [1,2]. Carotidynia can be associated with vascular processes (dissection, thrombosis, fibromuscular dysplasia, aneurysm, fibromuscular dysplasia, aneurysm, giant cell arteritis, or Takayasu arteritis) and also with non-vascular processes, such as lymphadenitis, sialadenitis, peritonsillar abscesses, and cervical neoplasms [2,3]. Clinically, this condition is characterized by localized cervical pain on the anterior side of the neck, which worsens with swallowing or coughing and may radiate to the face, jaw, or ear [1-3]. On physical examination, it presents as swelling or tenderness to palpation, and slight redness may also appear in the malar region [1,2,4]. It is a benign condition that can last from hours to weeks, averaging seven to 10 days. Given its inflammatory etiology, the common treatment for symptom resolution is the administration of nonsteroidal anti-inflammatory drugs (NSAIDs) or corticosteroids [4-6].

## Case presentation

**Figure 1:**
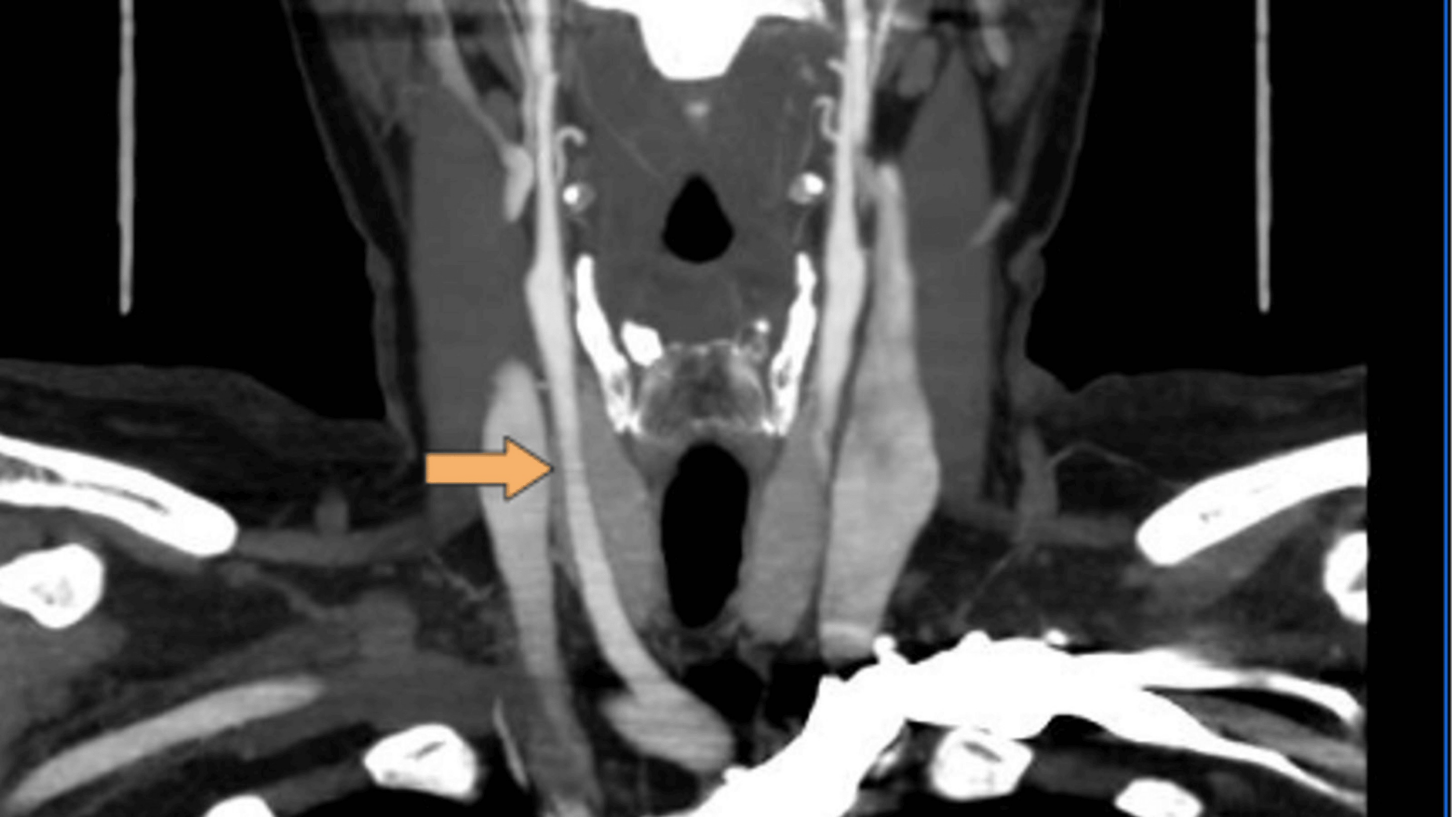
Initial CT angiography.

A 45-year-old female with a known history of eclampsia and heavy smoking was referred by her family physician for an internal medicine consultation due to the presence of a foreign body sensation in her right cervical region, with one week of evolution. During the physical examination, a tender right pulsatile mass without a thrill was identified. A cervical CT scan was performed, which revealed a calcified atherosclerotic plaque at the carotid bifurcation, as well as soft tissue thickening around the right internal carotid artery seen as a hypodense halo around it, thereby indicating unilateral adventitial thickening (Figure 1). To assess the vascular changes and exclude the presence of a dissection, a CT angiography of the neck and body was performed. The CT angiography results showed no changes in the patency of the various arterial paths, as well as no intraluminal filling defects. Given these findings, a course of naproxen was initiated, which resulted in symptom improvement.

An analytical and immunological study was also conducted prior to the naproxen treatment, revealing elevated results of erythrocyte sedimentation rate (ESR) and C-reactive protein (CRP), with a subsequent improvement after one month. The immunological study also detected high levels of anticardiolipin and anti-beta-2-glycoprotein (anti-B2-GPI) immunoglobulin M (IgM). These results were re-confirmed across two subsequent follow-up immunological studies.

Given these findings, 100 mg of aspirin and 200 mg of hydroxychloroquine, both once a day, were added to the treatment regimen, while 500 mg of naproxen twice a day was maintained over an indefinite period of time, at least a year.

**Figure 2:**
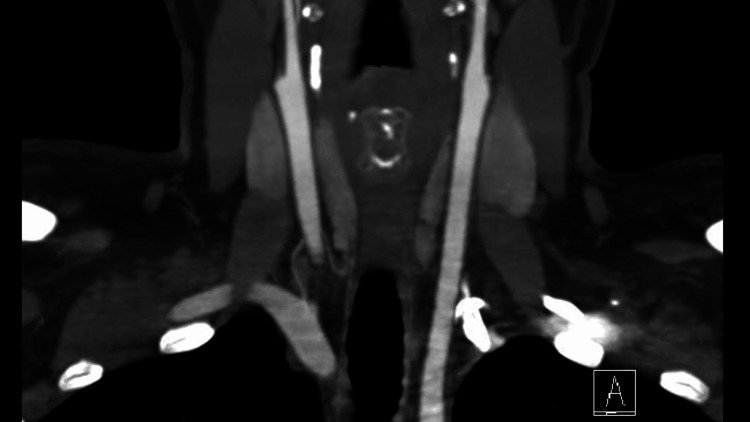
Follow-up CT angiography.

Three months later, a vascular control follow-up CT angiography showed improvement in the thickening around the right internal carotid artery (Figure 2). Four months later, a minor recurrence was observed, likely due to poor adherence to the treatment.

## Discussion

According to the IHS, the diagnosis of carotidynia must meet the following four criteria: (1) at least one of the following signs over the carotid artery: hypersensitivity, swelling, or increased pulsations; (2) appropriate investigation reveals no structural vessel changes; (3) pain over the affected side of the neck that projects to the ipsilateral side of the face; and (4) a self-limited syndrome lasting less than two weeks [7]. The evolution of diagnostic methods has allowed for a better characterization of the carotid region, defining carotidynia as a distinct pathological entity with structural changes and particular radiological features.

Its inflammatory etiology has gained strength over time. PET studies revealed an increased signal in the soft tissues around the carotid (foci of hypermetabolism), which, in the absence of a cervical mass and with a self-limited course, further supports the inflammatory nature of the lesion [8,9]. Additionally, levels of soluble intercellular adhesion molecule-1 (sICAM-1), a marker of large vessel vasculitis activity, as seen in giant cell arteritis and Kawasaki disease, are correlated with the clinical phase of carotidynia [5,8]. Evidence of signs of an active, low-activity chronic inflammatory process at the arterial wall becomes even stronger when treatment with NSAIDs and corticosteroids is implemented [6,8,10,11].

In this clinical case, the patient’s initial symptoms improved over time, and the thickening of the carotid wall, re-evaluated by CT angiography, also regressed. Among the limited studies on carotidynia, patient ages range from 16 to 55 years, with a higher prevalence observed in women. Given the strong inflammatory suspicion, recent scientific publications recommended the administration of corticosteroids or NSAIDs [1,5,6,8]. The administration of corticosteroids showed a more favorable clinical course, leading to complete remission of the structural changes [2].

However, in this specific case, the approach with naproxen was sufficient to resolve the symptoms and normalize the inflammatory parameters [2,8]. This case also involved the rare association of this condition with the presence of antiphospholipid antibodies, which led to the addition of aspirin and hydroxychloroquine to the treatment regimen with naproxen. Even considering the history of eclampsia, the patient did not meet the criteria for antiphospholipid syndrome (APS). She maintained irregular follow-up and variable adherence to the treatment and smoking cessation, eventually abandoning follow-up consultations.

## Conclusions

Carotidynia, or Fay syndrome, should be considered in the differential diagnosis of neck pain. A course of NSAIDs may be an effective treatment option, as spontaneous improvement of symptoms and complete remission of structural carotid wall abnormalities can occur within weeks. Recurrence of symptoms should be considered, particularly if external factors such as non-compliance with treatment are involved. This clinical case emphasizes the rare presence of antiphospholipid antibodies in a patient with carotidynia.
